# Supplementary material for: Causal interplay between lactose intolerance and gut microbiota: a combined bidirectional Mendelian randomization and in vivo validation study
Source: Front Nutr. 2026 Jun 1;13:1803337. doi: 10.3389/fnut.2026.1803337 (PMC13265576; doi:10.3389/fnut.2026.1803337)
Supplement: Supplementary file 1 [file Data_Sheet_1.zip › supplementary materials/Forward/leave one out cross validation/ebi-a-GCST90027697.finngen_R12_E4_LACTONAS.pdf]

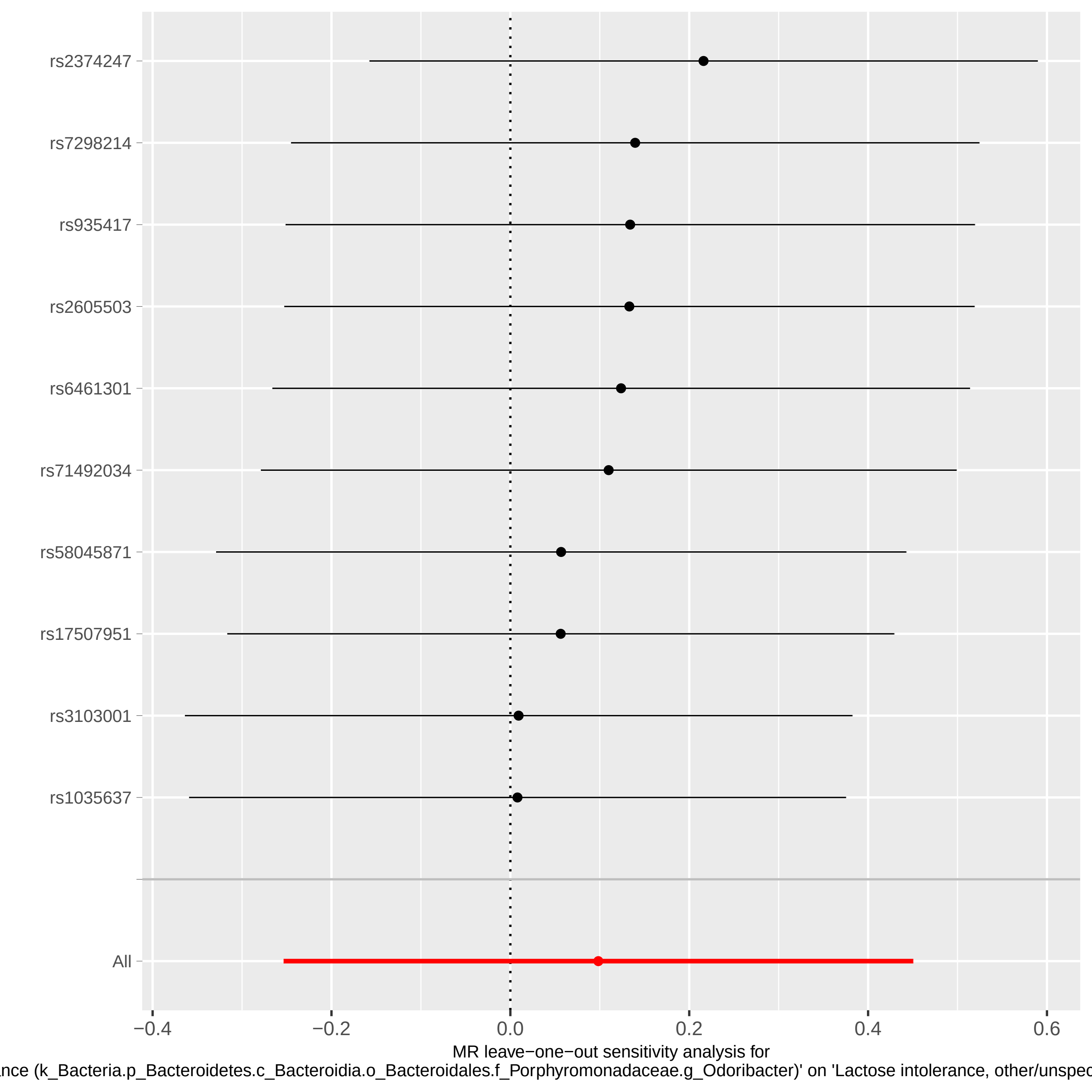
rs2374247
rs7298214
rs935417
rs2605503
rs6461301
rs71492034
rs58045871
rs17507951
rs3103001
rs1035637
All
−0.4
−0.2
0.0
0.2
0.4
0.6
MR leave−one−out sensitivity analysis for
nce (k_Bacteria.p_Bacteroidetes.c_Bacteroidia.o_Bacteroidales.f_Porphyromonadaceae.g_Odoribacter)' on 'Lactose intolerance, other/unspec
